# Life history linked to immune investment in developing amphibians

**DOI:** 10.1093/conphys/cow025

**Published:** 2016-08-26

**Authors:** Douglas C Woodhams, Sara C Bell, Laurent Bigler, Richard M Caprioli, Pierre Chaurand, Brianna A Lam, Laura K Reinert, Urs Stalder, Victoria M Vazquez, Klaus Schliep, Andreas Hertz, Louise A Rollins-Smith

**Affiliations:** 1 Department of Biology, University of Massachusetts Boston, 100 Morrissey Blvd., Boston, MA 02125, USA; 2 College of Marine and Environmental Sciences, James Cook University, Townsville, QLD 4811, Australia; 3 Department of Chemistry, University of Zürich, Winterthurerstrasse 190, CH-8057 Zürich, Switzerland; 4 Mass Spectrometry Research Center and Department of Biochemistry, Vanderbilt University, Nashville, TN 37232-8575, USA; 5 Department of Chemistry, Université de Montréal, Montreal, QC, Canada H3T 1J4; 6 Department of Biology, James Madison University, MSC 7801, Harrisonburg, VA 22807, USA; 7 Department of Pathology, Microbiology and Immunology, Vanderbilt University School of Medicine, Nashville, TN 37232-2363, USA; 8 Plant Biology Department, University of Georgia, Athens, GA 30602, USA; 9 Department of Biological Science, Vanderbilt University, Nashville, TN 37235-1634, USA; 10 Department of Pediatrics, Vanderbilt University School of Medicine, Nashville, TN 37232-2363, USA

**Keywords:** Antimicrobial peptides, disease ecology, innate immunity, life-history strategy, MALDI-TOF mass spectrometry, tadpoles

## Abstract

The broad diversity of amphibian developmental strategies has been shaped, in part, by pathogen pressure, yet trade-offs between the rate of larval development and immune investment remain poorly understood. The expression of antimicrobial peptides (AMPs) in skin secretions is a crucial defense against emerging amphibian pathogens and can also indirectly affect host defense by influencing the composition of skin microbiota. We examined the constitutive or induced expression of AMPs in 17 species at multiple life-history stages. We found that AMP defenses in tadpoles of species with short larval periods (fast pace of life) were reduced in comparison with species that overwinter as tadpoles and grow to a large size. A complete set of defensive peptides emerged soon after metamorphosis. These findings support the hypothesis that species with a slow pace of life invest energy in AMP production to resist potential pathogens encountered during the long larval period, whereas species with a fast pace of life trade this investment in defense for more rapid growth and development.

## Teaser

Amphibian population declines are often associated with disease outbreaks at metamorphosis. We examine the ontogeny of innate immunity in a range of amphibians and find that skin peptide defenses may partially compensate for adaptive immune suppression during this sensitive stage. Skin defense trade-offs with development reflect amphibian life-history strategy.

## Introduction

Recent ecological theory suggests that trade-offs exist between host defenses and pace of life (Martin *et al*., 2007; Previtali *et al*., 2012; Sandmeier and Tracy, 2014; Sears *et al*., 2015). In animals with complex life histories, such as amphibians, slow pace of life often refers to growth to a large size over a relatively long larval period, including overwintering as larvae in some species (Stoks *et al*., 2006; Johnson *et al*., 2012). Amphibians with a fast pace of life are more likely to use behavioural defenses against parasitic trematodes than slow pace-of-life species, and they appear to invest less in costly immune defenses that would provide infection tolerance (Sears *et al*., 2015). Here, we examine an innate immune defense of amphibians, antimicrobial skin peptides, in relation to amphibian life history and pace of life.

The cutaneous granular glands of many, but not all, amphibian species produce diverse gene-encoded antimicrobial peptides (AMPs) that are specific to species and populations (Apponyi *et al*., 2004; Woodhams *et al*., 2006a, b; 2010; Tennessen *et al*., 2009; Holden *et al*., 2015). Although peptide structure is clearly linked to amphibian phylogeny (Conlon *et al*., 2009b), peptide expression during ontogeny has not been carefully studied and may reflect ecological trade-offs between pace of life and immune investment (Todd, 2007). We hypothesized that slow pace-of-life species would produce and secrete effective AMPs during the larval period, whereas fast pace-of-life species would not.

Little is known about the ontogeny of amphibian skin peptide defenses. By northern blot analysis and *in situ* hybridization, mRNA for the two most abundant antimicrobial peptides (magainin and PGLa) was first detected at the beginning of metamorphic climax in *Xenopus laevis* tadpoles. When whole animals were homogenized, mature peptides could be isolated (Reilly *et al*., 1994). In a similar study of *Lithobates catesbeianus* tadpoles, expression of mRNA for ranalexin was not detected by northern blot analysis in premetamorphic tadpoles (forelimbs had not emerged), but was detected in metamorphosing tadpoles and adults (Clark *et al*., 1994). In both studies, production of AMPs was localized to developing cutaneous granular glands (Clark *et al*., 1994; Reilly *et al*., 1994). Katzenback *et al*. (2014) found increasing mRNA expression of brevinin-1SY through tadpole development in *Lithobates sylvaticus*. A study of *Rana ornataventris* adds to the evidence that late stage (metamorphosing) tadpoles of some species, but not early stage tadpoles, produce AMPs that are also expressed by adults. Temporin-1O was produced at the end of pre-metamorphosis and increased in late stage tadpoles and adults of this species (Iwamuro *et al*., 2006). Wabnitz *et al*. (1998) observed small amounts of host defense peptides in protein extracts from larval *Litoria splendida* as early as 14 days after egg deposition, but the complete set of adult skin peptides was not detected until metamorphosis. An ultrastructural study of *Phyllomedusa bicolor* indicated that both mucous and granular glands were present in tadpoles, but the gland duct did not appear to develop until metamorphosis (Lacombe *et al*., 2000). Using mass spectrometry (MS), known antimicrobial peptides were not consistently expressed in *Lithobates sphenocephalus* until ~12 weeks after metamorphosis (Holden *et al*., 2015). Although all of these studies provide some information about when in ontogeny AMPs can be expressed, they do not address the question of whether living tadpoles secrete defensive peptides onto the skin to function in microbial defense.

In this study, we used a combination of approaches to broadly assess the influence of host ontogeny and of interspecific variation in host life history on the expression of defensive skin peptides. Using matrix-assisted laser desorption time-of-flight (MALDI-TOF) MS, we tested for expression of AMPs in anuran species at developmental stages ranging from larvae to mature adults. In order to examine the influence of life-history trade-offs among species, we analysed AMP data from 17 anuran species and found that pace of life appears to influence immune investment. Our studies show that long-lived tadpoles of several species in two of six amphibian families sampled secrete a subset of the same active defensive peptides that are secreted by adults, but the full set of adult-type peptides does not emerge until metamorphosis. These studies also suggest that these innate immune defenses are an important aspect in the evolution of amphibian life-history strategy, and a trade-off exists between pace of life and investment in skin peptide defenses.

## Materials and methods

### Experimental amphibians

Seventeen species of anuran amphibians were examined in these studies. Sample sizes, life stages sampled and body size of all individuals are presented in Tables 1 and 2. Although sampling occurred at different field and laboratory sites, the sampling and collection methods were consistent across species.

**Table 1:** Skin peptide collections across anuran life-history stages

Species	Life-history stage (Gosner, 1960 stage)	*n*	Mass [g (mean ± SD)]	Method of skin peptide induction^a^	Quantity of peptides [μg/g body mass (mean ± SEM)]
*Alytes obstetricans*	Tadpole (25)	25^b^	0.30 ± 0.09	Bath	1297.56 ± 571.93
	Metamoprh	5	1.87 ± 0.18	Injection	2168.3 ± 410.4
	Adult	8	7.08 ± 0.88	Injection	574.87 ± 216.02
*Lithobates catesbeianus*	Tadpole (25–35)	7	21.99 ± 6.05	Bath	16.0 ± 3.3
	Tadpole (36–39)	9	38.50 ± 1.84	Bath	22.2 ± 10.8
	Tadpole (40–41)	9	44.18 ± 2.03	Bath	47.0 ± 15.7
	Tadpole (42)	2	18.60 ± 3.08	Bath	230.3 ± 101.0
	Metamorph	12	15.07 ± 2.76	Injection	66.4 ± 11.1
	Subadult	5	45.97 ± 19.51	Injection	256.5 ± 72.9
	Adult	10	100.1 ± 7.22	Injection	186.9 ± 61.4
	Adult	6	14.95 ± 2.09	Injection	459.8 ± 184.3
*Lithobates pipiens*	Tadpole (25)	9	2.44 ± 0.46	Bath	174.6 ± 8.5
	Metamorph	11	1.84 ± 0.62	Bath	247.7 ± 76.9
	Metamorph	3	2.44 **±** 0.76	Injection	248.5 ± 105.9
	Subadult	6	8.53 ± 2.42	Bath	64.5 ± 11.1
	Adult	13	30.38 ± 4.07	Injection	437.45 ± 68.1
	Adult	9	27.83 ± 8.54	Bath	42.7 ± 8.2
*Litoria serrata*	Tadpole (25)	107^c^	1.33 ± 0.71	Bath	105.0 ± 26.2
	Metamorph	8	0.25 ± 0.12	Bath	449.8 ± 131.9
	Adult	24	4.88 ± 1.18	Bath	31.2 ± 4.5
	Adult	20	4.06 ± 0.22	Injection	579.8 ± 50.5
*Rana sierrae*	Tadpole (25)	25^c^	1.32 ± 0.23	Bath	171.9 ± 16.8
	Metamorph	10	2.73 ± 0.44	Injection	1753.8 ± 596.0
	Adult	30	34.59 ± 14.38	Injection	636.1 ± 80.1
*Litoria nannotis*	Tadpole (25)	5	2.45 ± 0.43	Bath	240.3 ± 101.6
*Mixophyes shevilli*	Tadpole (25)	10	1.53 ± 0.41	Bath	77.3 ± 62.6
*Hyla cinerea*	Adult	10	6.91 ± 0.57	Injection	196.6 ± 42.1^d^

Secretions were induced by norepinephrine in the bath (tadpoles) or by subcutaneous injection (post-metamorphosis). Note that the peptide quantity is comparable across samples collected by the same induction method.

^a^Bath indicates immersion for 15 min in 100 µM norepinephrine bitartrate; injection indicates subcutaneous administration of 10 nmol/g body mass norepinephrine bitartrate.

^b^Twenty-five tadpoles; five groups of five tadpoles each.

^c^One hundred and seven tadpoles; 16 groups of 3–11 tadpoles each.

^d^No peptides were detected by mass spectrometry.

**Table 2:** Skin peptides detected in larval and post-metamorphic amphibians of 17 species

Species	Number of tadpoles examined (Gosner, 1960 stage)	Number of post-metamorphs examined	Proportion of tadpole:adult AMPs detected
*Alytes obstetricans*	25 (25)	13	0.88
*Bufo bufo*	30 (25)	15	0
*Bufotes viridis*	20 (25)	0	–
*Hyla meridionalis*	15 (37–38)	0	–
*Hyla versicolor*	4 (25)	0	–
*Lithobates catesbeianus*	27 (25–42)	33	0.73
*Lithobates pipiens*	9 (25–41)	42	0.14
*Litoria ewingii*	10 (Anstis, 2002; stage 41)	12	0
*Litoria serrata*	107 (25)	52	0
*Pelobates fuscus*	11 (25–40)	9	0
*Pelodytes punctatus*	12 (37–41)	3	0
*Pseudacris regilla*	4 (25)	0^a^	0
*Rana arvalis*	12 (25)	12	0
*Rana iberica*	11 (25–38)	4	0
*Rana sierrae*	25 (25)	40	0.25
*Rana temporaria*	60 (25)	13	0
*Rhinella marina*	0	3	0

Abbreviation: AMPs, antimicrobial peptides. Proportions are based on data presented in Table 3.

^a^*Pseudacris regilla* AMPs were detected by RNA analysis (Robertson & Cornman, 2014). *Litoria ewingii* data are from Schadich *et al*. (2010).

Tadpoles of northern leopard frogs, *Lithobates pipiens*, were obtained from a commercial supplier (NASCO, Fort Atkinson, WI, USA) and were maintained in groups of 10 in 16 litre tanks at Vanderbilt University. These and other species were sampled as tadpoles before limb development and as metamorphs immediately after tail resorption was complete. In July 2004, skin peptides from subadults were sampled in the field in Van Buren County, MI, USA. In September 2004, skin peptides from six adult *L. pipiens* were sampled from the same field location. Nine additional adult *L. pipiens* were obtained from Connecticut Valley Biological Supply Co., Southhampton, MA, USA, housed in 16 litre plastic containers and cared for as described below. Skin peptides from these frogs are described by Woodhams *et al*. (2006b) and Rollins-Smith *et al*. (2006).

Tadpoles of American bullfrogs, *Lithobates catesbeianus*, were sampled in the field in Boulder, CO, USA in June 2013. An egg mass was collected from Davidson County, TN, USA in June 2004, and tadpoles were reared in the laboratory at Vanderbilt University. Tadpoles and adults were also obtained from Charles D. Sullivan Co., Inc. Newly metamorphosed *L. catesbeianus* were obtained from a commercial supplier (Rana Ranch, Twin Falls, ID, USA). After 1 year, the subadults were sampled in the laboratory. Adult frogs were sampled in the laboratory in May 2007 at the University of Georgia.

At Sixty Lake Basin in the Sierra Nevada Mountains of CA, USA, skin peptides from adult mountain yellow-legged frogs, *Rana sierrae*, were collected in September 2004 (Rollins-Smith *et al*., 2006). At the same time and location, *R. sierrae* tadpoles were sampled for skin peptides. Metamorphs were sampled in the laboratory at James Madison University in January 2008 after being raised from an egg clutch salvaged from a drying pool in Sixty Lake Basin in the summer of 2007.

Green-eyed treefrogs, *Litoria serrata* (formerly *L. genimaculata*), were sampled for skin peptides at Birthday Creek, near Paluma, Queensland, Australia in September 2005 (adults) and December 2006 (tadpoles). Data were also included from Woodhams (2003), including skin peptide samples from metamorphs raised in the laboratory at James Cook University from tadpoles collected at Birthday Creek, Queensland, Australia in February 2003.

Common midwife toads, *Alytes obstetricans*, were raised at the University of Zurich through metamorphosis from egg clutches collected in Germany. Tadpoles and metamorphs were sampled for skin peptides in August 2008. Additional adults were sampled from Canton Basel, Switzerland in June 2009. Antimicrobial peptides were described by Conlon *et al*. (2009a).

The five species described above were sampled as tadpoles, as recent metamorphs and as juveniles or adults for comparisons across life history, examination of constitutive skin peptide expression and quantification of induced skin peptide secretions. An additional 12 species were examined to compare more broadly between larval and adult stages (Table 2). The following species were raised in Switzerland from field-collected egg clutches: *Bufo bufo* and *Rana temporaria* (Woodhams *et al*., 2014), *Bufotes viridis*, *Hyla meridionalis*, *Pelobates fuscus*, *Pelodytes punctatus*, *Rana arvalis* and *Rana iberica*. *Rhinella marina* were sampled in Panama, *Pseudacris regilla* were sampled in California, and *Hyla versicolor* were sampled from Minnesota. In addition to these field-sampled amphibians, data are included from *Litoria ewingii* sampled in New Zealand (Schadich *et al*., 2010). An additional three species were sampled from only one life stage and include *Litoria nannotis* and *Mixophyes shevilli* tadpoles sampled in the field in Queensland, Australia, and *Hyla cinerea* adults obtained from PETCO Animal Supplies, Inc. and sampled at the University of Colorado, Boulder, CO, USA.

### Animal care

Tadpoles were reared in dechlorinated tap water (changed twice weekly) and were fed boiled romaine lettuce. After forelimb emergence at stage 42 (Gosner, 1960), metamorphs were moved to 16 litre polystyrene containers set at an incline, with a small volume of water at one end so that the frogs could choose wet or dry conditions. All containers were sterilized with bleach and dried before use. Each newly metamorphosed juvenile was fed two or three vitamin-dusted crickets three times weekly.

### Skin peptide sampling

Table 1 lists the methods of skin peptide induction and sampling for MS analyses for each species and life-history stage. Granular gland secretions were induced from post-metamorphic amphibians by administration of norepinephrine (bitartrate salt; Sigma, St Louis, MO, USA) by immersion in an aqueous solution or by subcutaneous injection (Rollins-Smith *et al*., 2002; Woodhams *et al*., 2006b). Tadpoles were exposed to an aqueous solution of norepinephrine, and when sampled, all were at developmental stages between stages 25 and 41 (Gosner, 1960). Metamorphs were sampled immediately after the tail was completely resorbed. After administration of norepinephrine, skin secretions were collected, partly purified and quantified as previously described (Rollins-Smith *et al*., 2006). Dry weight was measured for all *A. obstetricans* peptides. The total quantity of peptides recovered per gram body weight was determined for each sample.

In addition to total skin secretion comparisons across life stages, the mixture of peptides locally present on different skin surfaces of adult frogs was sampled directly for MS analysis by applying 80 µm carbon-imbedded conductive polyethylene film (hereafter ‘peptide-absorbent film’; Goodfellow Cambridge Ltd, Cambridge, UK) as previously described (Woodhams *et al*., 2007). After norepinephrine induction of peptides in adult *L. pipiens* (*n* = 10), film was applied locally to the dorsal surface and dorsolateral ridge, ventral thigh and pelvic patch, ventral foot webbing and ventral gular skin for comparison of the relative intensity of peptide signals. Film was also applied locally to the dorsal and ventral surfaces of *R. sierrae* (*n* = 24) to detect constitutive expression of AMPs, and relative intensities were compared by Student's paired *t*-tests. Constitutive AMP expression from adults of several species has been described previously (Pask *et al*. 2012; Woodhams *et al*. 2012).

Tadpoles of all species were blotted with peptide-absorbent film across the body and tail before norepinephrine induction to determine whether peptide expression was constitutive or required induction. Thus, constitutively expressed (ambient) peptides were sampled from all tadpoles within seconds of capture, before stimulation. Tadpoles were then sampled as described above for induced peptide expression.

### Analysis of skin peptides by mass spectrometry

Skin peptide mixtures were analysed by MALDI-TOF MS following direct sampling with peptide-absorbent film (no purification or concentration steps) or after norepinephrine-induced peptide secretion, as previously described (Woodhams *et al*., 2007). Norepinephrine-induced peptide mixtures eluted from C-18 Sep-Paks were spotted onto the sample plate at 1 mg/ml before adding an equal volume of matrix. An Applied Biosystems Voyager DE-STR spectrometer was operated in reflector, delayed extraction and positive ion mode. For external calibration, a series of peptide standards (Sigma-Aldrich) was applied. Mass spectra were acquired across the range of *m*/*z* (mass to charge ratio) 600–10 000 and analysed after baseline subtraction and de-noising (smoothing) with Data Explorer v4.4 (Applied Biosystems). All samples from *A. obstetricans* were analysed by MALDI-TOF using an Autoflex I time-of-flight mass spectrometer (Bruker Daltonics GmbH, Bremen, Germany) equipped with a 337 nm nitrogen laser. A 20 µl sample solution was diluted with 20 µl of 0.1% trifluoroacetic acid, vortexed, and 1 µl was spotted onto a ‘Prespotted AnchorChip’ target prepared with α-cyano-4-hydroxycinnamic acid as matrix (CHCA; Bruker). Instrument calibration was obtained using signals from the HCCA matrix at *m*/*z* 379.09 and a mixture of standard peptides composed of bradykinin 1–7 (*m*/*z* 757.40), angiotensin II (*m*/*z* 1046.54), angiotensin I (*m*/*z* 1296.69), renin substrate (*m*/*z* 1758.93), adrenocorticotrophic hormone clip 18–39 (*m*/*z* 2465.20) and somatostatin 28 (*m*/*z* 3147.47), all obtained from the peptide calibration standard mix II (Bruker). Comparisons of peptide profiles were made among life-history stages of each species. Methods and MS–MS sequence confirmation of *Rana sierrae* tadpole AMP temporin-1M are provided in the Supplementary data as confirmation that the tadpole peptide has an identical structure to that found in adults. This peptide is also known to be antimicrobial and can inhibit the emerging pathogenic fungus *Batrachochytrium dendrobatidis* (*Bd*) at concentrations down to 6.25 μM (Rollins-Smith *et al*., 2006).

### Pace-of-life and immune function trade-offs

The ability to express defensive peptides at the tadpole stage was determined and this trait mapped onto a phylogenetic tree. Many of the peptides detected on tadpoles (e.g. Supplementary Fig. S1) were previously shown to inhibit bacteria and *Bd* (Rollins-Smith *et al*., 2006). We determined whether this trait was exclusive to the amphibian lineage, or appears among several lineages in association with pace of life. For the 17 anuran species examined here and in previous studies, we determined whether maximal tadpole size differed between species capable of expressing peptides as tadpoles, and between species that typically overwinter throughout their range by Student's unpaired *t*-test. We used Fisher's exact test to determine whether the proportion of species capable of expressing peptides as tadpoles differed between fast and slow pace of life. We corrected for phylogeny by using a phylogenetic independent contrast with the pic function in the ape package of R statistical software, in a similar manner to Johnson *et al*. (2012). We performed a linear regression and phylogenetically independent contrast analysis using the proportion of AMPs expressed as tadpoles as the dependent variable. The independent variable was a categorical variable indicating whether an amphibian species typically overwinters. To determine whether occurrence of an overwintering tadpole stage can predict the likelihood of AMP production at the tadpole stage, we used a binary logistic regression. Some genera, including *Bufo*, *Bufotes* and *Rhinella* (family Bufonidae), did not have detectable AMPs, and a limited number of AMPs from *Hyla* have been described (Table 2; Erspamer *et al*., 1986; Conlon, 2011). In a preliminary study, we did not detect skin peptides from adult *Hyla cinerea* by MS (Table 1), although secretions had antifungal activity (Woodhams DC, Rollins-Smith LA, Voyles J, and Carey C, unpublished data).

## Results

### Antimicrobial peptides detected by direct matrix-assisted laser desorption time-of-flight mass spectrometry

**Figure 1:**
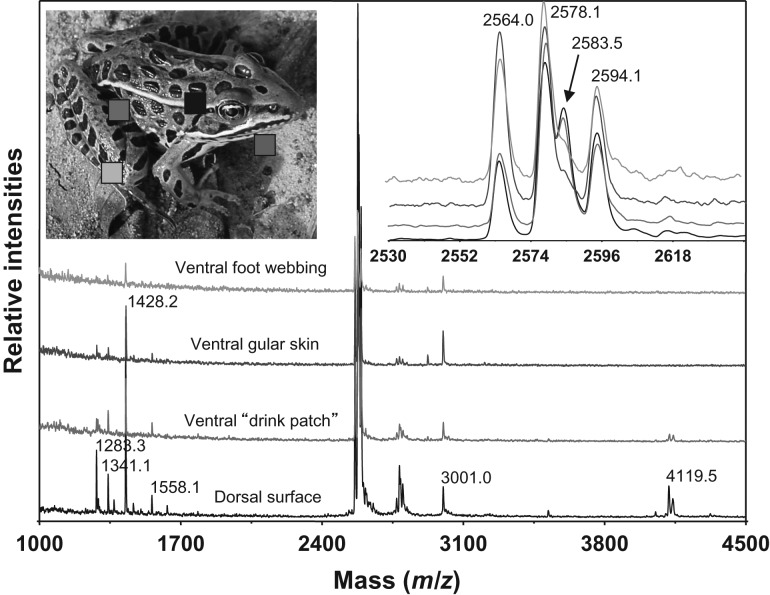
Peptide profiles from four skin surfaces of adult *Lithobates pipiens* upon induction of granular gland secretions. Representative spectra of 10 replicates are shown.

The expression of AMPs on the skin of live amphibians was detected by direct MALDI-TOF MS using samples from peptide-absorbent film blotted onto skin (Woodhams *et al*., 2007). Antimicrobial peptide signals were detected on all skin surfaces of adult *L. pipiens* induced with norepinephrine. The strongest relative intensity signals originated from films applied to the dorsal surface (Fig. 1). No significant differences were detected in the relative intensities of AMPs from dorsal or ventral surfaces of *R. sierrae* adults (temporin-1M, brevinin-1M, ranatuerin-2Ma and ranatuerin-2Mb; Student's paired *t*-tests, *P*-values > 0.05). The skin of all *R. sierrae* adults tested was heavily infected with the fungus *Bd* determined by qPCR (Briggs CJ & Vredenburg VT, unpublished data). In tadpoles, constitutive expression of peptides without norepinephrine induction was detected only in *L. catesbeianus*. Specifically, we observed mass signals for ranatuerin-2, -7, -8, -9 and ranalexin in this species.

### Induced peptides examined by matrix-assisted laser desorption time-of-flight mass spectrometry

**Figure 2:**
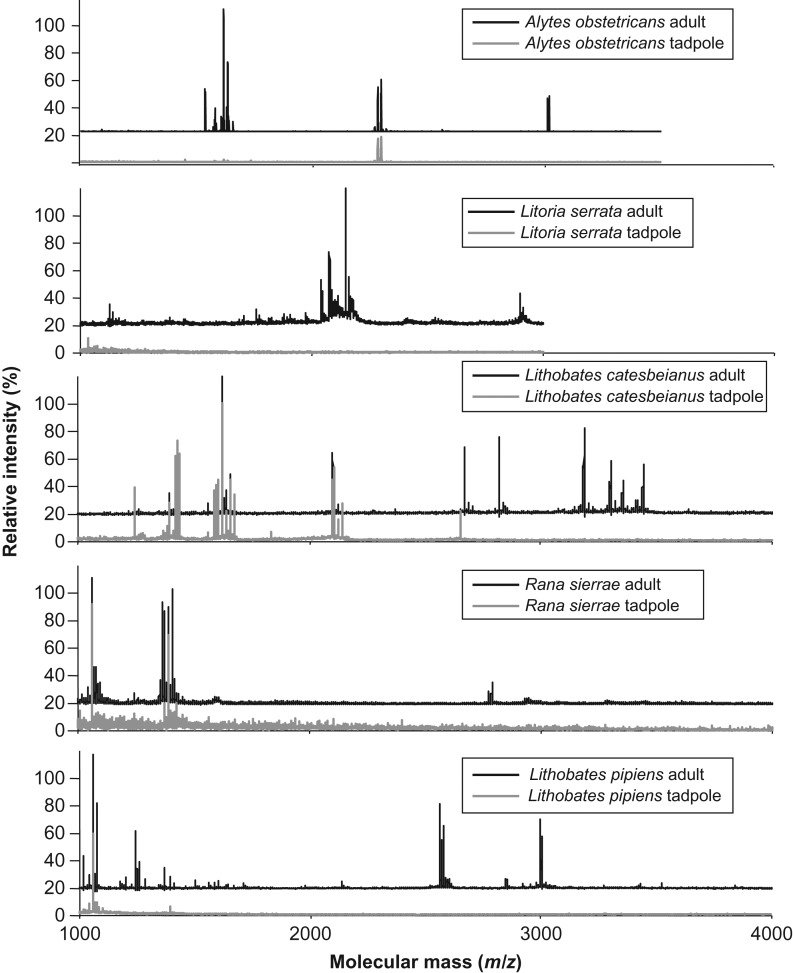
Representative skin peptide profiles of tadpoles and adults of five anuran species. Adult and metamorph spectra matched closely, and only adult profiles are displayed for clarity.

After peptide induction, mass signals indicative of previously described AMPs (Table 3) were detected in skin secretions from metamorphs and adults of all five species tested and from tadpoles of *A. obstetricans*, *L. catesbeianus*, *R. sierrae* and *L. pipiens* (Fig. 2). Nearly identical profiles were observed between adults and metamorphs of each species. A subset of the post-metamorphic peptides was detected in secretions from the tadpoles as shown in Fig. 2, and the specific peptides detected in each species are listed in Table 3 (adult peptide profiles are shown in comparison with those of tadpoles in Fig. 2). In *L. catesbeianus* tadpoles, ranatuerin-2, -4, -6, -7, -8 and -9, ranalexin and palustrin-2CBa were found. Bradykinin (non-antimicrobial) and temporin-1M or temporin-1P were found in the secretions of tadpoles of *R. sierrae* and *L. pipiens*. Of the eight skin peptides described for *A. obstetricans* (Conlon *et al*., 2009a), tadpoles expressed all except alyteserin-2c. The sequence structure of temporin-1M recovered from *R. sierrae* tadpoles was confirmed by MS–MS (Supplementary material, Fig. S1). Some peptides listed in Table 3 were not detected in the present study and might have been missing in the sampled individuals (Tennessen *et al*., 2009) or might not be detectable by MALDI-MS owing to poor ionization. In general, species with rapid larval development (*B. bufo*, *B. viridis*, *H. meridionalis*, *H. versicolor*, *L. pipiens*, *L. ewingii*, *L. serrata*, *P. regilla*, *R. arvalis*, *R. iberica*, *R. temporaria* and *R. marina*) showed few or no AMP signals typical of adults, suggestive of little investment in skin peptide defenses before metamorphosis (Table 2).

**Table 3:** Skin peptides detected from amphibian life-history stages by matrix-assisted laser desorption time-of-flight mass spectrometry (MALDI-TOF MS), with citations provided for peptide descriptions

Species	Skin peptide	Sequence	Mono-isotopic mass (*m*/*z*)	Signal detected by MALDI-TOF MS			Reference
				Tadpole	Metamorph	Adult	
*Alytes obstetricans*	Alytesin	pEGRLGTQWAVGHLM-NH2	1535.8	X	X	X	Erspamer *et al*. (1972)
	Alyteserin-2a	ILGKLLSTAAGLLSNL.NH2	1582.1	X	X	X	Conlon *et al*. (2009a)
	Alyteserin-2c	ILGAILPLVSGLLSSKL.NH2	1605.0		X	X	Conlon *et al*. (2009a)
	Alyteserin-2b	ILGAILPLVSGLLSNKL.NH2	1632.1	X	X	X	Conlon *et al*. (2009a)
	Alyteserin-1c	GLKEIFKAGLGSLVKGIAAHVAS.NH2	2263.5	X	X	X	Conlon *et al*. (2009a)
	Alyteserin-1a	GLKDIFKAGLGSLVKGIAAHVAN.NH2	2277.3	X	X	X	Conlon *et al*. (2009a)
	Alyteserin-1b	GLKEIFKAGLGSLVKGIAAHVAN.NH2	2291.4	X	X	X	Conlon *et al*. (2009a)
	Alyteserin-1d	GLKDIFKAGLGSLVKNIAAHVAN.NH2	2334.5	X	X	X	Conlon *et al*. (2009a)
*Lithobates catesbeianus*	Temporin-CBa (ranatuerin-5)	FLPIASLLGKYL.NH2	1333.8		X	X	Goraya *et al*. (1998); Hasunuma *et al*. (2010); Mechkarska *et al*. (2011)
	Temporin-CBf	FLPIASMLGKYL.NH2	1351.8				Mechkarska *et al*. (2011)
	Temporin-CBb (ranatuerin-6)	FISAIASMLGKFL.NH2	1396.8	X	X	X	Goraya *et al*. (1998); Rollins-Smith *et al*. (2002); Mechkarska *et al*. (2011)
	Ranatuerin-7	FLSAIASMLGKFL	1396.8	X	X	X	Goraya *et al*. (1998)
	Temporin-CBd (ranatuerin-8)	FISAIASFLGKFL.NH2	1412.8	X	X	X	Goraya *et al*. (1998); Mechkarska *et al*. (2011)
	Chensirin-2CBa	IIPLPLGYFAKKP	1455.9		X	X	Hasunuma *et al*. (2010)
	Ranatuerin-9	FLFPLITSFLSKVL	1624.0	X	X	X	Goraya *et al*. (1998)
	Brevinin-1CBa (ranalexin)	FLGGLIKIVPAMICAVTKKC	2104.2	X	X	X	Clark *et al*. (1994); Vignal *et al*. (1998); Rollins-Smith *et al*. (2002)
	Ranatuerin-1CBa (ranatuerin-1)	SMLSVLKNLGKVGLGFVACKINKQC	2649.5				Goraya *et al*. (1998); Rollins-Smith *et al*. (2002); Mechkarska *et al*. (2011)
	Brevinin-1CBb (ranatuerin-4)	FLPFIARLAAKVFPSIICSVTKKC	2651.5	X	X	X	Goraya *et al*. (1998); Mechkarska *et al*. (2011)
	Ranatuerin-1CBb	SMFSVLKNLGKVGLGFVACKVNKQC	2669.4		X	X	Mechkarska *et al*. (2011)
	Ranatuerin-2CBa (ranatuerin-2)	GLFLDTLKGAAKDVAGKLEGLKCKITGCKLP	3186.8	X	X	X	Goraya *et al*. (1998); Mechkarska *et al*. (2011)
	Palustrin-2CBa	GFLDIIKDTGKEFAVKILNNLKCKLAGGCPP	3301.8	X	X	X	Mechkarska *et al*. (2011)
	Ranatuerin-2CBc (ranatuerin-3)	GFLDIINKLGKTFAGHMLDKIKCTIGTCPPSP	3414.8		X	X	Goraya *et al*. (1998); Mechkarska *et al*. (2011)
	Ranatuerin-2CBd	GFLDIIKNLGKTFAGHMLDKIRCTIGTCPPSP	3442.8				Mechkarska *et al*. (2011)
*Lithobates pipiens*	Bradykinin	RPPGFSPFR	1059.6	X	X	X	Sin *et al*. (2008)
	Ranatensin-C	TPQWATGHFM	1174.5				Erspamer et al. (1986)
	Ranatensin-C	ZTPQWATGHFM	1303.2				Erspamer *et al*. (1984)
	Ranatensin	QVPQWAVGHFM	1298.6				Nakajima *et al*. (1970)
	Temporin-1P	FLPIVGKLLSGLL	1368.9	X	X	X	Goraya *et al*. (2000); Rollins-Smith *et al*. (2002)
	Peptide leucine arginine (pLR)	LVRGCWTKSYPPKPCFVR	2136.1				Salmon *et al*. (2001)
	Brevinin-1Pa	FLPIIAGVAAKVFPKIFCAISKKC	2563.5		X	X	Goraya *et al*. (2000)
	Brevinin-1Pd	FLPIIASVAANVFSKIFCAISKKC	2569.4		X	X	Goraya *et al*. (2000)
	Brevinin-1Pb	FLPIIAGIAAKVFPKIFCAISKKC	2577.5		X	X	Goraya *et al*. (2000)
	Brevinin-1Pc	FLPIIASVAAKVFSKIFCAISKKC	2583.5		X	X	Goraya *et al*. (2000)
	Brevinin-1Pe	FLPIIASVAAKVFPKIFCAISKKC	2593.5		X	X	Goraya *et al*. (2000)
	Ranatuerin-2P	GLMDTVKNVAKNLAGHMLDKLKCKITGC	3000.6		X	X	Goraya *et al*. (2000); Rollins-Smith *et al*. (2002); Chen *et al*. (2003)
	Ranatuerin-2Pa	GFLSTVVKLATNVAGTVIDTIKCKVTGGCRK	3178.8				Chen *et al*. (2003); Vanhoye *et al*. (2003)
	Esculentin-2P	GFSSIFRGVAKFASKGLGKDLARLGVNLVACKISKQC	3868.1				Goraya *et al*. (2000); Rollins-Smith *et al*. (2002)
*Litoria serrata*	Caerulein	QQDYTGWMDF	1290.5				Rozek *et al*. (1998)
	Maculatin-2.1	GFVDFLKKVAGTIANVVT	1878.1		X	X	Rozek *et al*. (1998)
	Maculatin-1.1.1	FGVLAKVAAHVVPAIAEHF	1975.1		X	X	Rozek *et al*. (1998)
	Maculatin-1.1	GLFGVLAKVAAHVVPAIAEHF	2145.2		X	X	Rozek *et al*. (1998)
	Maculatin-1.2	GLFGVLAKVASHVVPAIAEHFQA	2360.3				Rozek *et al*. (1998)
	Maculatin 3.1	GLLQTIKEKLESLESLAKGIVSGIQA	2723.6		X	X	Rozek *et al*. (1998)
							Rozek *et al*. (1998)
*Rana sierrae*	Bradykinin	RPPGFSPFR	1060.6	X	X	X	Rollins-Smith *et al*. (2006)
	Temporin-1M	FLPIVGKLLSGLL.NH_2_	1368.9	X	X	X	Rollins-Smith *et al*. (2006)
	Temporin-1M (free acid)	FLPIVGKLLSGLL	1369.9		X	X	Rollins-Smith *et al*. (2006)
	Ranatuerin-2Mb	GIMDSVKGVAKNLAAKLLEKLKCKITGC	2929.6		X	X	Rollins-Smith *et al*. (2006)
	Ranatuerin-2Ma	GLLSSFKGVAKGVAKDLAGKLLEKLKCKITGC	3273.9		X	X	Rollins-Smith *et al*. (2006)

### Pace-of-life and immune function trade-offs

**Figure 3:**
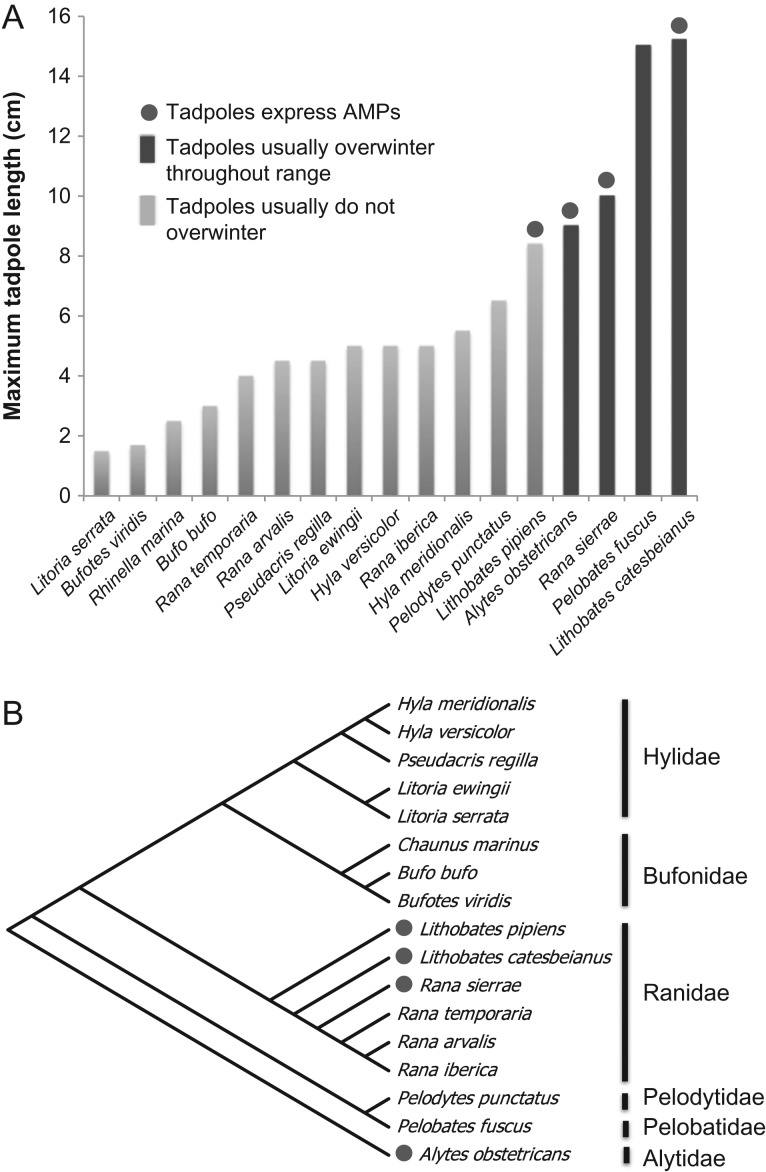
Species sampled and tadpole characteristics. (**A**) Maximal tadpole length as reported in species descriptions (AmphibiaWeb, 2016). Species with large tadpoles that tend to overwinter and are categorized here as ‘slow pace-of-life’ species (dark blue). (**B**) Neighbour-joining tree of taxa tested in this study. The analysis involved 29 nucleotide sequences of the 16S rRNA gene. The final data set consists of 439 aligned nucleotide positions. Distances were computed using the p-distance method and are in the units of the number of base differences per site. Analyses were conducted in MEGA7 (Kumar *et al*., 2016). Species with tadpoles expressing AMPs (green circles) are not exclusively found within a single family.

Of the 17 species examined in this analysis, four grow to a large size, develop slowly and usually overwinter as tadpoles (Fig. 3A). One species that can overwinter, *P. fuscus*, did not produce detectable AMPs in the tadpole stage, but peptides were detected by the same method in metamorphs. The other three overwintering species did produce detectable AMPs as tadpoles. Tadpoles that usually overwinter throughout their range reach a larger maximal size than those of typically non-overwintering species (Fig. 3A; Student's unpaired *t*-test, *t*_15_ = 6.143, *P* < 0.001). Likewise, species with tadpoles capable of expressing skin AMPs reach a significantly larger size as tadpoles than species that did not express skin AMPs as tadpoles (Fig. 3A; Student's unpaired *t*-test, *t*_15_ = 3.021, *P* = 0.009). A significantly higher proportion of slow pace-of-life species (three of four) were found to express peptides as tadpoles compared with fast pace-of-life species (one of 10; Table 3; Fisher's exact test, *P* = 0.041). The proportion of the AMP repertoire expressed as tadpoles in comparison with adults is on average 0.011 in non-overwintering species and 0.464 in overwintering species (Mann–Whitney *U*-test, *P* = 0.032). This characteristic is not exclusive to a single amphibian family (Fig. 3B), and a phylogenetic independent contrast using the pic function in the ape package of R statistical software showed that the trait of peptide expression at the tadpole stage differs by pace of life even after phylogenetic correction (*F*_1,15_ = 10.69, *P* = 0.00517). Thus, we propose that an overwintering tadpole stage predicts the likelihood that a species will produce AMPs as a tadpole (binary logistic regression, *P* = 0.021, odds ratio = 36).

## Discussion

### Evolution of amphibian developmental strategies

An amazing diversity of reproductive and developmental strategies exists among amphibian species that have implications for immune function and disease resistance (Todd, 2007; Gomez-Mestre *et al*., 2012). For example, some species have the capacity to overwinter in an aquatic tadpole stage, typically growing slowly to a large body size and requiring more permanent bodies of water. This may lead to exposure to a broad range of pathogenic organisms and greater resource allocation into immune defenses (e.g. Johnson *et al*., 2012). Other species, such as *L. serrata* and *L. pipiens*, can develop quickly in ephemeral water bodies or slow-moving creeks and may therefore devote fewer resources to immune function during the tadpole stage. Our data provide further support for the hypothesis of a trade-off between rapid growth and investment in immune defenses. We compare both among species that differ in life-history strategy and between developmental stages within a subset of those species.

Relative to adult frogs, tadpoles either completely lacked or only expressed a reduced set of defensive skin peptides (Fig. 2). This was particularly evident in rapidly developing species that appear to invest little in tadpole AMP defense (Table 3). We did not detect AMPs in tadpoles of 12 species with short larval periods or in one species, *P. fuscus*, capable of overwintering in the larval stage. In contrast, tadpoles with a long larval period (*A. obstetricans*, *L. catesbeianus* and *R. sierrae*) expressed a subset of adult AMPs with known capacities for inhibiting amphibian pathogens, including *Bd* (Rollins-Smith and Conlon, 2005). Among 17 species, those with long-lived larval stages were most likely to produce AMPs (Table 2), suggesting that slower pace-of-life species invest more resources in AMP skin defense during the larval stage than fast pace-of-life species. As we did not test for overall immune function, it is possible that fast pace-of-life species have comparatively weak immune defenses or rely on alternative defenses during larval development, such as microbiota, that may be less energetically expensive (Kueneman *et al*., 2014). Although our sampling here of a few populations from either field or laboratory settings represents a broad preliminary survey, we expect that further studies may refine these results by testing members of additional families or across a variety of environmental conditions and populations among species.

Some authors suggest that diseases and parasites (pathogenic fungi in particular; Green, 1999) are overlooked when explaining the diversity of amphibian developmental strategies (Todd, 2007). This may apply to trade-off strategies within species that have resulted in the evolution of flexible metamorphic timing. The timing of metamorphosis varies widely depending on larval conditions, including aquatic habitat (ephemeral or permanent), predation, density, competition, nutrition, pollutants and other factors (Smith-Gill and Berven, 1979; Werner, 1986; Alford and Harris, 1988). Developmental strategies may optimize growth in the least risky environment (Pechenik, 2006; Scott *et al*., 2007). In general, our data support the broad hypothesis that there may be a trade-off between larval growth and development of immune function, specifically AMP defenses. Studies by Groner *et al*. (2014) provide some support for this hypothesis, showing adjustments in skin peptide defense investment depending on stressors experienced early in ontogeny.

### Localized skin peptide expression

We found antimicrobial peptides constitutively present on both dorsal and ventral surfaces of *R. sierrae*, and upon induction, AMPs were detected on all skin surfaces of adult *L. pipiens* (Fig. 1). A previous study showed that the amount of peptides recovered from resting and active (chased) *L. pipiens* was sufficient to inhibit *Bd* (Pask *et al*., 2012). Given that the strongest signals detected by direct MALDI-TOF on *L. pipiens* were associated with the dorsal surface, other skin surfaces may be slightly more prone to infection. The legs and feet of some amphibians have been shown to be most prone to infection by *Bd* (North and Alford, 2008), and this may also be influenced by the ontogeny of keratinized skin, developing first in the feet and hindlimbs at metamorphosis, or because these surfaces come into contact with the contaminated substrate more often than other skin surfaces (Marantelli *et al*., 2004; Weldon and Du Preez, 2006).

There are several processes by which AMPs can be excreted. Granular glands of amphibian skin may not be able to discharge their AMP products fully onto the skin surface by a holocrine process until after development of the neuromuscular secretory apparatus and gland ducts in the epidermis (Delfino, 1980; Faszewski and Kaltenbach, 1995; Delfino *et al*., 1998, 2006; Lacombe *et al*., 2000). However, mature gland products may also be secreted by a merocrine process (either constitutive or induced exocytosis) and flow between epidermal cell layers and through epidermal interstices onto the skin surface before complete gland duct development (Delfino *et al*., 1998; Terreni *et al*., 2003; Quagliata *et al*., 2006). Some studies used transmission electron microscopy to examine discharge of granular glands (Delfino *et al*., 2006). Other studies used northern blotting, *in situ* hybridization or RT-PCR to detect mRNAs encoding AMPs (Clark *et al*., 1994; Reilly *et al*., 1994; Iwamuro *et al*., 2006; Katzenback *et al*., 2014). As none of these studies demonstrated secretion of active peptides from living tadpoles, we chose to use direct MALDI-TOF MS to examine AMP expression on tadpoles (Chaurand *et al*., 1999; Woodhams *et al*., 2007). By this method, we were able to detect constitutive expression of multiple AMP signals from the skin of *L. catesbeianus* tadpoles.

### Other components of amphibian skin defense

The mucous layer covering amphibian skin is an ideal niche for many opportunistic pathogens because it contains mucopolysaccharides that are a potential nutrient source. Hence, defense of the skin is crucial for protection from many amphibian pathogens and involves both adaptive and innate immune defenses, including mucosal antibodies, epithelial barriers, phagocytic cells, AMPs, fatty acids, protease inhibitors and other factors (Carey *et al*., 1999; Rollins-Smith *et al*., 2009; Ramsey *et al*. 2010). In addition, symbiotic microbes can contribute to skin defense and may also produce antimicrobial metabolites (Brucker *et al*., 2008; Harris *et al*., 2009). Some of these components of amphibian immune defense may be interacting synergistically or antagonistically with AMPs during metamorphosis (Myers *et al*., 2012; Woodhams *et al*., 2014). Changes in skin defenses during ontogeny, as shown here, may partly explain corresponding shifts in skin microbiota (Kueneman *et al*., 2014, 2016; Krynak *et al*., 2015).

### Disease susceptibility at metamorphosis

Several studies suggest that chytridiomycosis and other diseases can be most severe as amphibians undergo metamorphosis (Green *et al*., 2002; Bosch and Martínez-Solano, 2006; Carey *et al*., 2006; Garcia *et al*., 2006; Kriger and Hero, 2006; Rachowicz *et al*., 2006; Langhammer *et al*., 2014) or before they mature into adults (Abu Bakar *et al*., 2016). Experimentally reducing skin peptides in juvenile *X. laevis* caused increased susceptibility to *Bd* (Ramsey *et al*., 2010) and resulted in lethal chytridiomycosis in new metamorphs of *L. pipiens* (Pask *et al*., 2012). The mouthparts of larval amphibians can be infected by *Bd*, but tadpoles are largely immune to chytridiomycosis (Berger *et al*., 1998; Rachowicz and Vredenburg, 2004). The fungal pathogen *Bd* is thought to use keratin in tadpole mouthparts and adult amphibian skin. Upon metamorphosis, the fungus can spread from the infected mouthparts to the keratinized skin and lead to rapid mortality (Marantelli *et al*., 2004; Rachowicz and Vredenburg, 2004). Pathogenicity factors, including proteases that may degrade AMPs, are increased by exposure of *Bd* to thyroid hormone, which peaks during metamorphosis (Thekkiniath *et al*., 2013, 2015). Antimicrobial skin peptides could theoretically play a vital role in protecting some amphibians during this sensitive stage of development. However, if the new metamorphs experienced stress, such as limited food conditions, prior to metamorphosis, they might have fewer stored AMPs in granular glands available for defense. This might explain why newly metamorphosed *L. sphenocephala* raised in outdoor mesocosms were slow to develop an adult pattern of AMPs (Holden *et al*., 2015). Population level variation, not measured in this study, may also lead to variation in disease susceptibility at metamorphosis (Tobler and Schmidt, 2010; Bradley *et al*., 2015).

The composition of peptides differed among life-history stages and species (Tables 2 and 3). Such variation in skin peptide defense provides a potential mechanism for differential colonization by microbiota among species and life stages (McKenzie *et al*., 2012; Kueneman *et al*., 2014, 2016; Krynak *et al*., 2015) and susceptibility to infection from a variety of pathogens leading to disease or malformation. A holistic measure of the mucosome, or the combined host- and microbiota-derived compounds in the mucus, can test function against pathogens and predict disease susceptibility (Woodhams *et al*., 2014). Many of the diseases impacting amphibian populations are transmitted by pathogens or parasites in the aquatic environment that interact with the skin mucosome, including ranavirus, *Aeromonas hydrophila*, *Bd*, *Saprolegnia ferax*, *Anurofeca richardsi*, *Amphibiocystidium* spp. and *Ribeiroia ondatrae*. Although the Gram-negative bacterium *A. hydrophila* is not inhibited by amphibian skin peptides tested to date (Rollins-Smith *et al*., 2002; Schadich and Cole, 2009; Tennessen *et al*., 2009), both ranaviruses and *Bd* can be inhibited (Rollins-Smith *et al*., 2002; Chinchar *et al*., 2004). The ability of tadpole peptides to inhibit other bacterial pathogens, protozoa or fungal infections, such as *S. ferax* (Romansic *et al*., 2006), remains to be tested. The contribution of larval skin defenses to differences among species in infection by the malformation-inducing trematode *R. ondatrae* (Johnson and Hartson, 2009) is also unknown. *Lithobates catesbeianus* is significantly more resistant to *R. ondatrae* infection than species such as *P. regilla* and *B. americanus*, which lack AMP defenses as tadpoles (Table 2; Johnson *et al*., 2013; Calhoun *et al*., 2016). Skin peptides tested here do not appear to explain resistance of *H. versicolor* and other *Hyla* species to *R. ondatrae* (Johnson and Hartson, 2009; LaFonte and Johnson, 2013).

Ecologically, metamorphosis is a particularly vulnerable time for amphibians. Locomotion ability at metamorphosis lags below that of both tadpoles and adults, leaving metamorphs at greater risk of predation (Wassersug and Sperry, 1977). The type of predation, competition and environmental conditions, in addition to infection status, influence size at metamorphosis (Alford, 1999; Parris and Cornelius, 2004; Vonesh and Warkentin, 2006; Groner *et al*., 2014), and size at metamorphosis may influence subsequent survival (Pechenik, 2006; Scott *et al*., 2007). The timing of metamorphosis and stress during early ontogeny may have a significant influence on disease risk (Rollins-Smith, 1998; Groner *et al*., 2014). Pace of life appears to trade off with AMP immune investment in amphibian larvae. The repertoire of defensive peptides expressed on the skin changes with amphibian development such that at most a subset of the adult peptides occurs in tadpoles. Immunologically, the adaptive immune system reorganizes during metamorphosis, and innate skin peptide defenses may compensate for adaptive immune suppression, allow for restructuring of the microbiota upon metamorphosis and alter colonization resistance of parasites and pathogens.

## Supplementary material

Supplementary material is available at *Conservation Physiology* online.

## Supplementary Material

Supplementary Data
