# Supplementary figures and images for: Life history linked to immune investment in developing amphibians
Source: Conserv Physiol. 2016 Aug 26;4(1):cow025. doi: 10.1093/conphys/cow025 (PMC5001151; doi:10.1093/conphys/cow025)

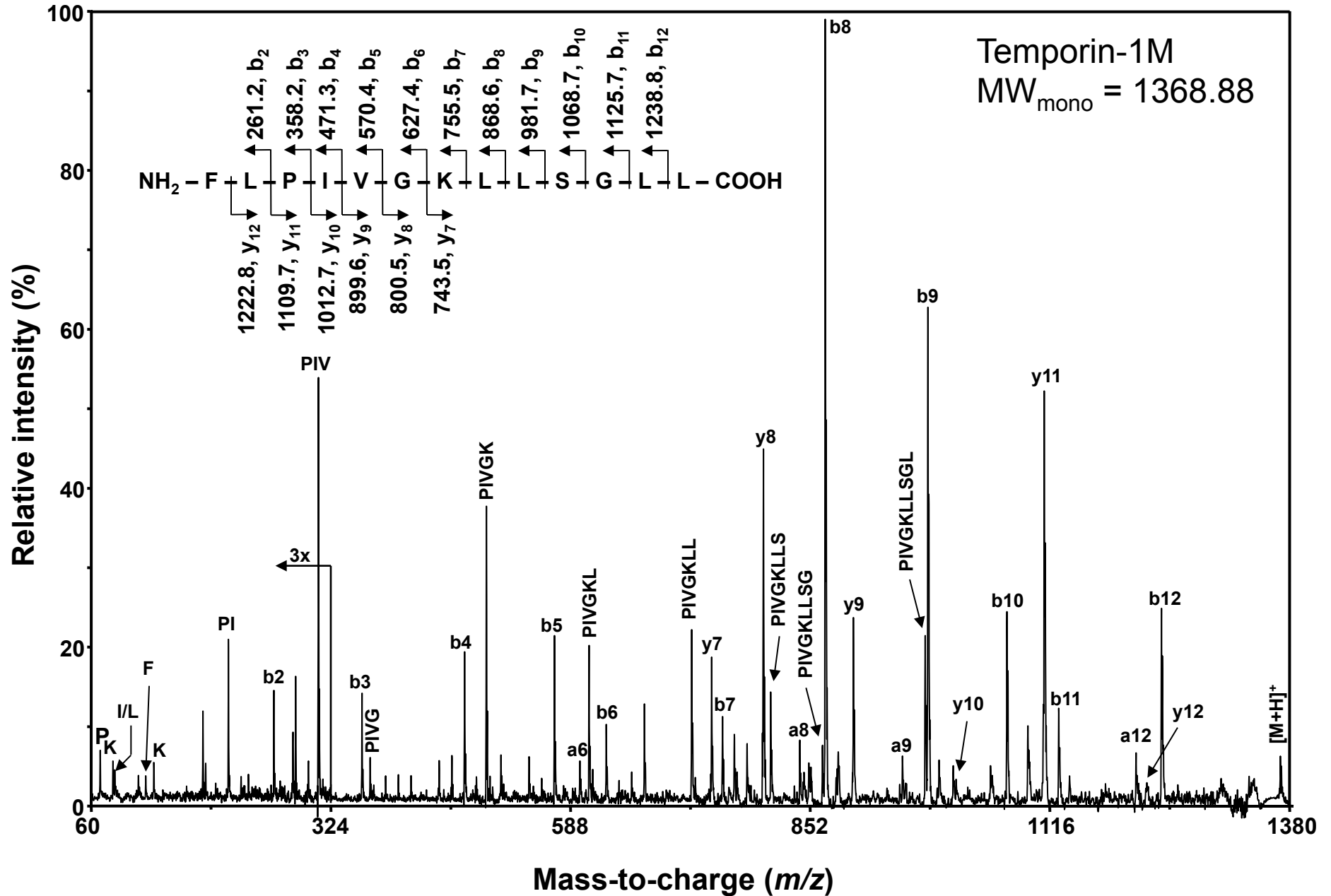

Supplement: Supplementary Data [file cow025_supplemental_figure_1.pdf]
